# Reirradiation treatment effects in the clinic (ReTEC) proposal – proof of concept based on spinal cord dose tolerance for reirradiation with stereotactic body radiotherapy

**DOI:** 10.1002/acm2.70557

**Published:** 2026-04-09

**Authors:** Jimm Grimm, Anand Mahadevan, Olusola Obayomi‐Davies, Scott G. Soltys, Arjun Sahgal, Kristin J. Redmond, Michael T. Milano, Wolfgang A. Tomé, Kenneth Brooks, Scott Dodd, John Austin Vargo, Gopal Subedi, Jinyu Xue, Bisher Ebid, Felix Ehret, Byung‐Han Andrew Rhieu, Christoph Fürweger, Rachel Grimm, Alexander Muacevic, Vitali Moiseenko, Shalom Kalnicki, Mark McLaughlin, Lawrence R. Kleinberg, John R. Adler, Iris C. Gibbs

**Affiliations:** ^1^ Wellstar Health System Marietta Georgia USA; ^2^ Department of Medical Imaging and Radiation Sciences Thomas Jefferson University Philadelphia, PA USA; ^3^ NYU Langone Health New York New York USA; ^4^ Stanford University Stanford California USA; ^5^ Sunnybrook Health Science Centre University of Toronto Toronto Ontario Canada; ^6^ Johns Hopkins University Baltimore Maryland USA; ^7^ University of Rochester Medical Center Rochester New York USA; ^8^ Montefiore Medical Center and Albert Einstein College of Medicine Bronx New York USA; ^9^ UPMC Magee‐Women's Hospital Pittsburgh Pennsylvania USA; ^10^ Georgia Institute of Technology Atlanta Georgia USA; ^11^ Department of Radiation Oncology Charité – Universitätsmedizin Berlin Corporate Member of Freie Universität Berlin and Humboldt‐Universität zu Berlin Berlin Germany; ^12^ German Cancer Consortium (DKTK), partner site Berlin a partnership between DKFZ and Charité – Universitätsmedizin Berlin Berlin Germany; ^13^ European Radiosurgery Center Munich Munich Germany; ^14^ University of California San Diego La Jolla California USA

**Keywords:** Reirradiation, Recovery Factors, Spinal Cord

## Abstract

**Background:**

Since the inception of spinal radiosurgery decades ago, the factors influencing spinal cord recovery have remained unclear. Elucidating these factors would aid in reirradiation planning and delivery. We describe modeling reirradiation normal tissue complication probabilities (NTCP) from compiled published data, akin to the collaborative Quantitative Analysis of Normal Tissue Effects in the Clinic (QUANTEC) and High Dose per Fraction, Hypofractionated Treatment Effects in the Clinic (HyTEC) projects.

**Purpose:**

Simple dose‐time recovery factors for the spinal cord serve as a basis to propose the Reirradiation Treatment Effects in the Clinic (ReTEC) project. ReTEC aims to define human organ recovery factors to convert the total composite dose to an equivalent physical dose compatible with the de novo therapy dose tolerance limits. Additionally, we present a system for automating analyses as well as clinical implementation of radiation dose‐time recovery.

**Methods:**

Published literature was queried for stereotactic voxel‐wise composite reirradiation plans involving the spinal cord, but none were found. Hence, minimum reporting criteria were used instead: spinal cord maximum dose (Dmax) of each course, time interval between courses, and number of fractions or biologically effective dose, per patient or in sufficiently homogeneous groups of patients. Recovery factors and logistic model parameters were fitted with maximum likelihood techniques.

**Results:**

An NTCP model was derived from 13 papers with data from 282 lesions, including six myelopathy cases. The model suggested spinal cord recovery of more than 50% in less than 1 year, which is faster and more complete than current clinical practice, but with wide confidence intervals (CI) (bootstrap 95% CI: 31%–95% when the time interval was 1 year).

**Conclusion:**

Due to limited data, we recommend remaining within current standard guidelines until more comprehensive analyses, optimally under the ReTEC initiative, refute or validate these estimates. ReTEC would require composite plans accounting for differing Dmax locations in each course. Nevertheless, this proof‐of‐concept Dmax model provides insight and serves as a basis to propose ReTEC.

## 1 INTRODUCTION

Reirradiation of spinal tumors after prior conventional or stereotactic radiosurgery is increasingly utilized for recurrence of spine metastases, but more information is needed to define normal tissue dose tolerance based on recovery time after radiation, as well as the specifics of dosimetry of both treatment courses. More than a decade ago, when dose tolerances for organs at risk (OARs) were uncertain for stereotactic body radiation therapy (SBRT), or stereotactic ablative body radiotherapy (SABR), a comprehensive review on this topic was published in this journal^1^ to propose the High Dose per Fraction, Hypofractionated Treatment Effects in the Clinic (HyTEC) project.^2^, ^3^ HyTEC focused not only on normal tissue complication probabilities (NTCP), but also on modeling tumor control probabilities (TCP) based upon published outcome data. The previously mentioned review, which proposed HyTEC, became the third most downloaded article from this journal (only surpassed by American Association of Physicists in Medicine (AAPM) practice guideline reports),^4^ emphasizing the interest and its utility for clinicians in moving the field forward.

Here we use the available data to embark upon another emerging area of study that would greatly benefit from dose response modeling: Reirradiation is currently in a stage of rapid clinical adoption, with growing interest in recent years highlighted by efforts for standardization and reporting,^5^ but without clear guidelines for implementation. Even though the first publication on spine radiosurgery consisted entirely of reirradiation cases,^6^ it was not until recently that sufficient details have been published to enable a spinal cord recovery model to be analyzed from clinical data. Given the proximity of the spinal cord, “definitive” irradiation of tumors of the vertebral column has always been challenging, until precise stereotactic delivery approaches were developed capable of giving high dose treatment adjacent to the spinal cord with rapid dose falloff. Given that spine metastases are common after treatment of many types of primary tumors, it was understood that SBRT might serve an important role in the reirradiation of spinal metastasis. In fact, many of the earliest reports of SBRT for spinal metastases were in the setting of tumor recurrence following previous conventional radiation therapy.^6^, ^7^, ^8^ Although practiced for many decades, detailed published data on reirradiation is only now burgeoning.

Currently, dose‐time recovery factors, including the residual dose effect (RDE) for specific OARs such as the spinal cord, are poorly understood. There is a need to develop a methodology that will enable conversion of the total reirradiation dose to a biological equivalent compatible with existing dose tolerance limits. However, given that dose tolerance in the setting of reirradiation remains highly uncertain, we propose the Reirradiation Treatment Effects in the Clinic (ReTEC) project. The objectives of the project are to systematically analyze published data in an attempt to understand the effects of dose and recovery time on tissue toxicities, thereby refining dose‐time recovery factors in the reirradiation setting.

Hence, instead of compiling yet another list of new dose tolerance limits for reirradiation, we pivot to define simple dose‐time recovery factors that will allow conversion of the total composite dose into a biologically effective dose (BED) compatible with de novo dosing, to enable dose volume histograms (DVH) evaluation of composite plans with reference to existing limits for anatomical organs throughout the body, in most clinical circumstances. As NTCP in the setting of reirradiation is a nascent discipline with limited reporting in the literature, we were not able to identify a single complete dataset with full 3D voxel‐wise composite dose distributions of each course, the time interval between courses of irradiation, and follow‐up outcomes for the spinal cord. Consequently, this paper merely proposes an avenue to achieve such conversion, with a preliminary spinal cord recovery model for maximum point dose (Dmax) as a proof‐of‐concept example. The new model is compared to existing reirradiation guidelines,^3^, ^9^ and a system for automating the analysis, as well as clinical implementation of the radiation dose‐time recovery, is presented. The results of our analysis highlight the need for additional outcome data with improved reporting standards: organ dose distribution, fractionation, toxicity outcomes for each course, and the time interval between each course, for each patient.

## 2 METHODS

A simple reirradiation recovery model for dose distribution to the OAR is defined as

(1) $$Dose_{Sum}=Dose_{Prior}*RDE+Dose_{ReTx},$$

where $Dose_{Sum}$ is the time discounted cumulative dose with a goal of being biologically equivalent to a de novo treatment, $Dose_{Prior}$ is the previously treated dose, *RDE* is the time varying residual dose effect, and $Dose_{ReTx}$ is the current reirradiation treatment. Our goal is to refine the models to the point where $Dose_{Sum}$ can be compared to existing de novo dose tolerance limits. However, it is important to note that this represents a proof‐of‐concept model to propose the ReTEC project, so the goal of full compatibility with de novo models has not yet been achieved.

The *RDE* could be intuitively modeled as an exponential function, but it is known that late effects can occur many years later, so radiation must have some maximum recovery (*R_max_*) that is less than unity, indicating there is a long‐term residual effect equal to 1‐*R_max_*. Therefore, we model this portion as a horizontal line, which is added to raise the exponential function as:

(2) $$RDE=1-R_{max}\left(1-e^{-kt}\right),$$

where the constant *k* is to ensure the total model goes through 50% recovery when $t=t_{RH}$, where $t_{RH}$ denotes the recovery half time, which yields:

(3) $$k=\frac{1}{t_{\mathrm{RH}}}\;\ln\left[R_{\max}/\left(R_{\max}-1/2\right)\right].$$

If any organs do not recover as much as 50%, then Equation (3) cannot be used, but Equation (2) may still be used with *k* as a general parameter. However, for organs that do have at least 50% recovery, it will be helpful to know the overall $t_{RH}$. Note that the *R_max_* is attained asymptotically at an infinite time interval, but for all practical purposes, this may be assumed to have been reached when the time interval between treatments is 3 to 4 times $t_{RH}$ or more. The amount of recovery at any given time instant is 1‐*RDE*, since residual is the opposite of recovery. In general, if the prior radiation sensitizes instead of recovers, *R_max_* would be negative and *RDE* would be greater than unity.

PubMed searches using the dates and terms specified in Appendix A were used to identify studies that would allow one to glean data from the published literature, as summarized in the subsequent Figures and Table 1 in this manuscript. The inclusion criteria for data used in the model are the following minimum patient‐specific reporting standards: prior spinal cord maximum dose or reasonably accurate nominal surrogate thereof,^16^ reirradiation spinal cord maximum dose, time interval between courses, fractionation or BED, utilization of SBRT for at least one of the courses, and myelopathy outcomes in the absence of progression. Dosimetric and outcome analyses on sacral plexus and brainstem were excluded; only data on the spinal cord proper and cauda equina were included in the model. In the proposed ReTEC project, the data will include full 3D dose distributions with the number of fractions for each course of each patient to allow for BED parameter optimization. However, in this proof‐of‐concept endeavor, it was necessary to also include studies that only provided the Dmax BED. Most of the source data manuscripts used the linear quadratic (LQ) model with *α*/*β* = 2 Gy for the spinal cord (BED_2_), so we used this to convert all data to BED_2_ prior to any other modeling. The recovery factors and logistic model parameters were optimized in the DVH Evaluator software (DiversiLabs, LLC, Bloomsburg, Pa) with maximum likelihood (ML) parameter estimation^23^ of the logistic model.^24^ The maximum likelihood parameter searches were performed only up to a *R_max_* of 95%, given that late effects can occur in the spinal cord even many years after treatment completion. Statistical significance was assessed via the likelihood ratio test (LRT). In all NTCP graphs throughout this manuscript and Appendices, a solid blue line represents the logistic model within the range of existing data for that graph, and the dashed blue lines indicate modeled values at doses above or below the available data. Green dashed lines are profile likelihood confidence intervals (CI) assuming *t_RH_* and *R_max_* were known, whereas black dash‐dot lines are bootstrap CI accounting for the uncertainty of all modeling parameters.

**TABLE 1:** Reirradiation spinal cord studies that provided initial organ dose, time interval, reirradiation organ dose, and myelopathy outcome.

	Spinal cord	Time interval	Spinal cord			Non‐sacrum
	Dmax crs1	crs1 to crs2, crs3 ^a^	Dmax crs2, crs3 ^a^	Follow‐up		Cord+Cauda
References	(BED_2_, Gy)	(Months)	(BED_2_, Gy)	(Months)	Myelopathy	NumCases
Gwak 2005^10^	95.76	54	85.90	32		1
Gwak 2005^10^	75	120	120.90	24		1
Gerszten 2006^11^	75	3	40	4		1
Parikh 2009^12^	75	6	77.34	26		1
Choi 2010^13^	80	19	49.5	7		7 ^b^
Choi 2010^13^	80	19	84.99	7		10 ^b^
Choi 2010^13^	80	19	82.88	7		5 ^b^
Choi 2010^13^	80	19	76.13	7		3 ^b^
Choi 2010^13^	80	19	73.13	7		12 ^b^
Damast 2011^14^	75	25	33.6	12.1		35 ^b^, ^c^, ^d^
Nikolajek 2011^15^	85.6	15	40	14.5		53 ^b^, ^c^
Nikolajek 2011^15^	85.6	8.8	40	14.5	1	1
Sahgal 2012^16^	60	5	20.14	9		1
Sahgal 2012^16^	103	61	3.71	26		1
Sahgal 2012^16^	101	7	14.38	12		1
Sahgal 2012^16^	86	62	97.63	35		1
Sahgal 2012^16^	86	85	35.35	31		1
Sahgal 2012^16^	72	20	75.99	3		1
Sahgal 2012^16^	75	19	21.22	48		1
Sahgal 2012^16^	81.44	15, 7 ^a^	24, 117.9 ^a^	21		1
Sahgal 2012^16^	68	32	25.09	27		1
Sahgal 2012^16^	72	36	31.68	11		1
Sahgal 2012^16^	75	5	23.03	18		1
Sahgal 2012^16^	75	5	23.85	18		1
Sahgal 2012^16^	84	5	17.00	4		1
Sahgal 2012^16^	129	39	52.34	7		1
Sahgal 2012^16^	75	6	25.03	5		1
Sahgal 2012^16^	100	11	56.85	8		1
Sahgal 2012^8^, ^13^, ^16^	76	81	123.32	55	1	1
Sahgal 2012^8^, ^16^	37	70	130.10	29	1	1
Sahgal 2012^16^	66	11	87.95	17	1	1
Sahgal 2012^10^, ^16^	100	18	209.73	11	1	1
Sahgal 2012^16^	105	12	122.75	3	1	1
Chang 2012^17^	74.36	24.5	92.38	17.3		37 ^b^, ^c^, ^e^
Wang 2014^18^	80	9	117.2	9.4		12 ^b^
Thibault 2015^19^	66.8	14.3	47	6.8		24 ^b^, ^c^
Thibault 2015^19^	75	12.9, 14.3 ^a^	39.4, 49 ^a^	6.8		17 ^b^, ^c^
Zschaeck 2017^20^	90	18, 8 ^a^	127.6, 70.8 ^a^	12		1
Zschaeck 2017^20^	96	61, 11 ^a^	107, 45.6 ^a^	12		1
Ehret 2021^21^	79.2	17.2	103.18	22.2		38 ^b^
Bentahila 2023^22^	69.4	101, 85 ^a^	5.78, 7.88 ^a^	8		1

Abbreviations: Dmax = maximum dose; crs = course; Gy = Gray; BED_2_ = biologically effective dose with α/β = 2 Gy; NumCases = number of cases.

^a^ Five datapoints had 3 courses, and those have the separate values for each course in this column, separated by a comma.

^b^ NumCases has been reduced to exclude local failures from the analysis.

^c^ NumCases has been reduced to exclude the sacrum from the analysis.

^d^ Only patients from the high‐dose arm were included in this SBRT analysis.

^e^ Prior spinal cord dose was approximated as total spinal cord dose minus the reirradiation spinal cord dose.

## 3 RESULTS

**FIGURE 1:**
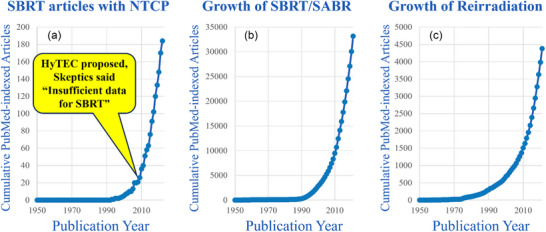
Comparison of growth of journal articles on NTCP for SBRT and reirradiation based on the PubMed searches in Appendix A. (A) At the time HyTEC was proposed, only about 20 NTCP papers were found, but about 90% of the current NTCP for SBRT models were created during the project. Therefore, this type of data shortage should not prevent the start of the ReTEC project. (B),(C) The trajectory of about 5000 reirradiation papers now is similar to the 5000 SBRT publications at the beginning of the HyTEC project, and those have continued to be published prolifically. NTCP = normal tissue complication probabilities; SBRT = stereotactic body radiation therapy; SABR = stereotactic ablative body radiotherapy; HyTEC = High Dose per Fraction, Hypofractionated Treatment Effects in the Clinic; ReTEC = Reirradiation Treatment Effects in the Clinic.

Searches of PubMed (Appendix A) show that publications for reirradiation have rapidly increased since the 1990s and a similar rate of increase is demonstrated in SBRT utilization trends, as shown in Figure 1. Only 20 articles that include NTCP models for SBRT were identified in the publication search through the year 2009 (Figure 1A), but there are almost 200 now. Figure 1B illustrates the growth in SBRT publications from about 5000 in 2009 to a cumulative number of about 35,000 by 2020, and Figure 1C shows that reirradiation publications have grown to about 5000 now.

**FIGURE 2:**
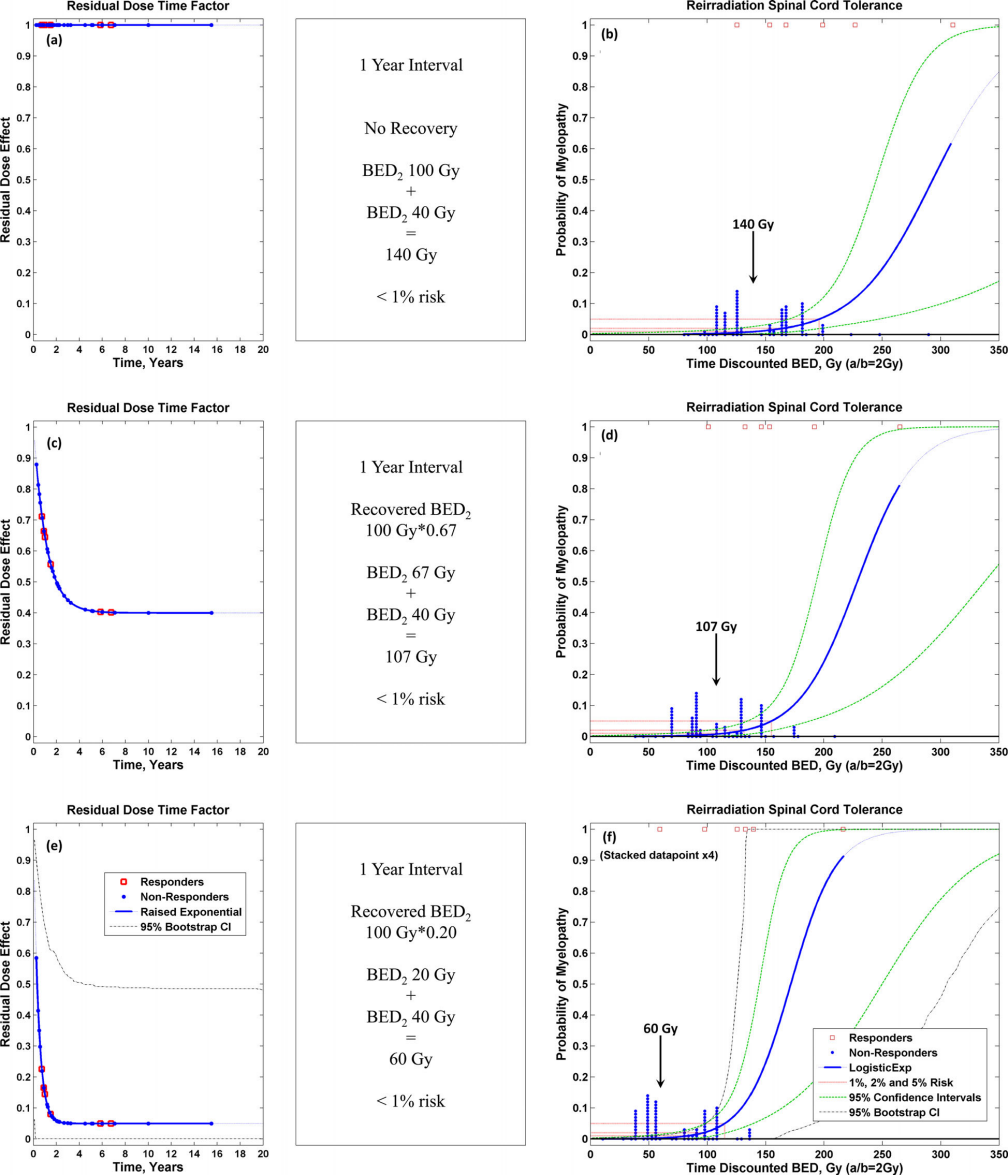
(a,b). Scenario 1 of 3: Recovery model, clinical example, and dose‐response model for the scenario of no recovery. The example case of spinal cord Dmax of 50 Gy in 25 fractions followed by reirradiation with spinal cord Dmax of 15.5 Gy in five fractions in the same location a year later, with no recovery, would have composite BED_2_ = 140 Gy as denoted by the arrow, which corresponds to less than 1% risk according to the model. (c,d). Scenario 2 of 3: Recovery model, clinical example, and dose‐response model for the scenario of Nelson 2009,^9^ 33% recovery after 1 year. Applying the time‐discounted recovery to every patient and refitting the dose response brought the cumulative spinal cord Dmax of the example patient down to BED_2_ = 107 Gy. The corresponding risk is still just below 1% because the recovery model was applied to every patient before refitting the dose response, and since the example case is near the centroid of the data. (e,f). Scenario 3 of 3: Recovery model, clinical example, and dose‐response for the scenario of maximum likelihood fitted *t_RH_* = 4 months and *R_max_* = 95%, which yields a time‐discounted composite spinal cord Dmax of BED_2_ = 60 Gy for the example case. As in Figure 2(b) and 2(d), the corresponding risk for the example case is still just less than 1%, because the recovery model was applied to all patients and then the dose response was refitted, and since the example case is near the centroid of the data. BED_2_ = biologically effective dose with α/β = 2 Gy; Gy = Gray; *t_RH_* = recovery halftime; *R_max_* = recovery maximum; Exp = exponential.

**FIGURE 3:**
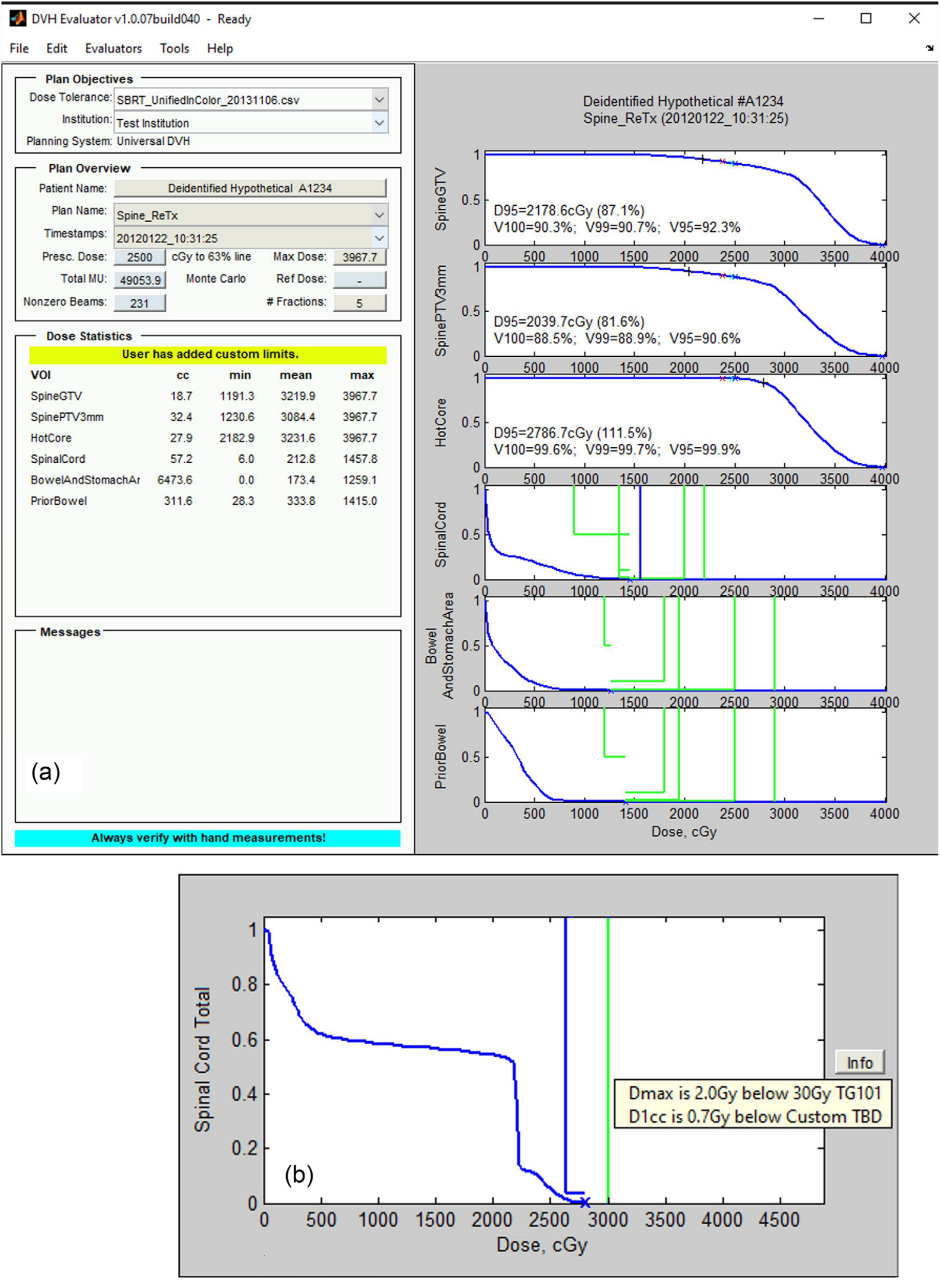
Clinical deidentified example of a spinal reirradiation case in the DVH Evaluator software. The first course of 45 Gy in 25 fractions homogeneously to thoracic vertebrae T2 through T10 with spinal cord Dmax = 91.5 Gy BED_2_, was followed by 25 Gy in five fractions a year later to the one vertebral body that relapsed. (A) Each DVH of the reirradiation course is shown on its own axis with user‐selectable limits overlaid to ensure dose tolerance limits are met. Even though the maximum likelihood model in Figure 2(e,f) estimates much faster and more complete recovery, a conservative 33% recovery was still applied,^9^ because the model still needs more thorough clinical validation from future studies with full composite plans. (B) Total spinal cord dose including the time‐discounted prior plus the reirradiation had a five‐fraction equivalent Dmax = 28 Gy, shown in the DVH Evaluator, compliant with the de novo AAPM TG‐101^25^ Dmax limit of 30 Gy in 5 fractions. Volume effects of reirradiation recovery are beyond the scope of this manuscript, so a custom D1cc limit is shown “to be determined” as a topic for future work. DVH = dose volume histogram; BED_2_ = biologically effective dose with α/β = 2 Gy; Gy = Gray; SBRT = stereotactic body radiation therapy; ReTx = reirradiation treatment; GTV = gross tumor volume; PTV = planning tumor volume; cGy = centi‐Gray; Dmax = maximum dose; D1cc = minimum dose to the “hottest” one cubic centimeter of spinal cord, TBD = to be determined.

Using the available data, we present preliminary models in the following four figures, in terms of BED_2_ from the LQ model with *α*/*β* = 2 Gy. The highest prior dose scenario from Table 4 of the HyTEC spinal cord paper^3^ is used as an example in Figures 2, 3, with a 50 Gy in 25 fractions (BED_2 _= 100 Gy) prior irradiation followed by reirradiation with spinal cord Dmax limit of 15.5 Gy in 5 fractions (BED_2 _= 40 Gy) in the same location, assuming a 1‐year time interval between courses. Figure 2(a,b) presents the reirradiation model with the most conservative treatment assumption of “no recovery”. In this model, the 1% probability of spinal cord myelopathy is associated with a composite dose of approximately 140 Gy without time‐discounting the BED_2_. However, it is essential to note that this dose value is specific to this model, which assumes “no recovery”. Alternatively, Figure 2(c,d) shows the reirradiation model with a 1‐year time interval using 33% recovery of the initial treatment proposed by Nelson et al.^9^ In this model, it is noted that 1% probability of spinal cord myelopathy occurs at about 107 Gy for the time‐discounted BED_2_, showing that model parameters affect the estimated cumulative dose. Finally, Figure 2(e,f) shows the maximum likelihood fitted values (*p* < 0.01, LRT) with a *t_RH_* of 4 months and *R_max_* of 95% for the 1‐year time interval. In this model, it is noted that 1% probability of spinal cord myelopathy is near just 60 Gy for the time‐discounted BED_2_, which is dramatically different than the non‐recovery scenario. The example case of spinal cord *D_max_* of 50 Gy in 25 fractions followed by 15.5 Gy in 5 fractions after 12 months has less than 1% risk in all three scenarios, underscoring the importance of ensuring that the correctly associated recovery factors are applied with the chosen model. Testing the effect of a wide range of possible *t_RH_* and *R_max_* values, as in these three scenarios, serves as a preliminary type of sensitivity analysis, and one of the key goals of the proposed ReTEC project will be to determine how accurately *t_RH_* and *R_max_* can be estimated with the best attainable data, and at that time assessing the impact of potential residual uncertainty of *t_RH_* and *R_max_* on the clinical outcomes.

For clinical illustration, an example patient was chosen who was treated to thoracic levels T2‐T10 with conventional fractionation, and a year later was reirradiated to T2‐T3 with SBRT. The initial course had a spinal cord Dmax 4.3% higher than the prescription. To explore the effects of the recovery factors at different dose levels, the same plan was scaled into two separate example cases: (A) 50 Gy in 25 fractions followed by SBRT with spinal cord Dmax of 14.58 Gy in five fractions a year later (see Table A1 and Figure A2), and (B) re‐scaled as if the prior dose was 30 Gy in 10 fractions, which would permit higher SBRT spinal cord Dmax of 17.9 Gy in five fractions enabling a higher prescription (see Table A1 and Figure A3).

Example A is illustrated clinically in the DVH Evaluator software in Figure 3, using the Nelson 2009 values^9^ for the recovery model, for clinical conservatism. The initial 50 Gy in 25 fractions plan to thoracic vertebral bodies T2‐T10 had spinal cord Dmax = 52.1 Gy (106.3 Gy BED_2_), which is similar to the 100 Gy BED_2_ prior dose example of Figure 2, but also including the fact that in conventional planning, the spinal cord Dmax may be up to about 5% above the prescribed dose. For clinical convenience, the composite BED values including the 33% recovery factor are converted to a five‐fraction equivalent dose, to compare with reference to existing de novo limits. Figure 3(A) shows the reirradiation plan a year later, 25 Gy in five fractions with maximum spinal cord below the 15.5 Gy/5 fraction Sahgal 2021 reirradiation limit,^3^ and (B) shows the composite spinal cord DVH with the 33% recovery per Figure 2(c,d). In this example, the composite spinal cord Dmax dose converted to five‐fraction equivalent dose is still compatible with the AAPM TG‐101 limit of 30 Gy in five fractions.^25^ Since human reirradiation volume effects data were not yet published but are a major goal of the proposed ReTEC effort, a D1cc volume limit^1^ was plotted conceptually in Figure 3 without a numerical limit.

Examples A and B show that the HyTEC spinal cord Dmax Tables 3 and 4^3^ are compatible with each other when the time interval between courses is 12 months or more per Nelson 2009 assumptions,^9^ including an allowance for conventional spinal cord Dmax being up to 5% above prescription (details in Appendix A). The ML recovery model of Figure 2(e,f) shows compatibility at 5–6 months as well. These guidelines have been used clinically for more than a decade after they originated from Sahgal 2012,^16^ so we are optimistic that the improved models from the ReTEC project will find validation, but we will not know for certain until the end of the project.

## 4 DISCUSSION

Given the potential for severe morbidity, radiation‐induced myelopathy is one of the most feared complications in radiation oncology. Despite generations of clinical experience, we still have difficulty knowing how to assess risk from spine reirradiation. In part, this challenge stems (fortunately) from the relative rarity of radiation‐caused spinal cord injury, which (unfortunately) limits the amount of available clinical experience to draw quantifiable conclusions. In turn, the rarity of complications cannot be separated from the near ubiquitous tendency to treat paraspinal lesions with radiation doses that are innately grounded in “an abundance of caution”. This overall lack of patient injury data to analyze makes it that much harder to know where the “edge of the ‘complication’ cliff” might lie. Meanwhile, radiation methods have changed substantially in recent years with the introduction of image‐guided stereotactic delivery, thereby lessening the extent of spinal cord irradiation and altering the radiation “volume effect”, inherently reducing the low‐dose “bath” to large volumes, which may enable a higher “shower” dose to smaller volumes.^26^ The issue of defining spinal cord dose tolerance is particularly difficult to unravel in the situation when the patient's spinal cord has already received prior radiation. Ultimately, as systemic therapies improve, cancer patients are undeniably living longer, and by virtue of such are more likely to confront multiple cancer relapses, some of which will be near a previously irradiated spinal cord. Because of the gravity of any decision in such patients, it has never been more important to quantitatively better elucidate factors governing the risk of spinal cord injury from ionizing radiation.

Our literature search (Figure 1A–C) showed a dramatic increase in both reirradiation and SBRT since the 1990s, with the current growth of reirradiation papers similar to the SBRT publications at the beginning of the HyTEC project in 2009. At that time, only 20 articles were identified that included NTCP models of SBRT (Figure 1A), and many experts did not think it was possible to conduct a dose‐response modeling project like Quantitative Analysis of Normal Tissue Effects in the Clinic (QUANTEC)^27^ on NTCP for SBRT^3^, ^28^ because of the lack of data. However, approximately 90% of the currently existing NTCP models for SBRT were published during the HyTEC project^3^ from 2009 to 2021. At the beginning of the HyTEC project, there were about 5000 papers on SBRT, and now it has grown to about 35000 papers (Figure 1B). Currently, we have found about 5000 papers on reirradiation, and the graphs in Figure 1B,C show a similar trajectory. Based on comparing the time trajectory of those previous projects, we expect usable data to emerge soon; therefore, this seems like the right time to embark on such a project for reirradiation.

Therefore, we present three separate NTCP models of myelopathy using data for reirradiation of the spinal cord in Figure 2 and an example of clinical implementation in Figure 3. Application of any model for patients must be done with a complete set of parameters applied in the same order as the original model was constructed. The addition of recovery factors (i.e., restoration of dose tolerance over time since the prior radiotherapy) makes it all the more important to apply the entire model in the proper sequence. For example, if the “no recovery assumption” was applied to the example patient's data but the maximally likelihood recovery factor NTCP model was used, the 140 Gy composite dose applied to the model in Figure 2(e,f) would erroneously estimate a 27% risk of myelopathy, which would be entirely wrong. Conversely, if the maximally likelihood recovery factor was applied to the example patient's data but the “no recovery” NTCP model was used, the 60 Gy composite dose applied to the model in Figure 2 would estimate less than 0.001% risk, which is incorrect in the other direction, and may lead to risky treatment. However, when the full set of parameters is applied in the correct order, the example case has about 1% risk for any of the recovery models in Figure 2.

Our model in Figure 2 consists entirely of human data, but since it is the first of its kind, it is important to compare it to other published results, as in Appendices B‐D, including animal models. Appendix B includes source code for equations (2), (3) and compares the raised exponential recovery model of equations ((1), (2), (3)) to the Gompertzian model, Appendix C shows fitting of the raised exponential model to the Nelson 2009 values,^9^ and Appendix D uses the newly designed raised exponential recovery model to re‐analyze existing pre‐clinical animal studies. Similar models have been applied to animal data for many decades, as described in Appendix D (refD01‐refD02, refD04‐refD05) and in the Discussion,^29^ but to our knowledge, this is the first human reirradiation recovery model for the spinal cord. Highlighting the specifics pertinent to reirradiation and modeling, two key aspects of the study by Ang et al. (refD04) are particularly important to discuss. Firstly, the analysis, including recovery, is done completely in terms of physical dose, without any BED conversions. Ideally, it would be preferable if the models could be refined to the extent that all analyses could be in terms of physical dose in each fractionation. However, much of the available spinal cord reirradiation data is only in terms of BED_2_ without fractionation being reported per patient. Therefore, it is critical that future publications include full details regarding dose/fractionation schedules per patient. Secondly, recovery factors were computed as compared to a de novo NTCP model where “all of the curves were required to be parallel at the 50% incidence level” (refD04), which is a method of helping to ensure the recovery model will be compatible with de novo dose tolerance limits.

### 4.1 Importance of remaining within dose tolerance limits and recommended time intervals

Spinal cord dose tolerance is one of the most strictly respected OAR limits in radiation oncology. The currently available published clinical data are within this safe range, so no attempts were made for the current model to include the effects of exceeding dose tolerance during the initial course of radiotherapy. A preclinical model^29^ includes a prior dose term (1 – *PercentageOfToleranceDose*/100), implying zero recovery if tolerance was exactly reached and negative recovery if tolerance was already exceeded. Such a factor is an important warning and should be investigated when complete datasets become available, but the model in this manuscript only uses the human data from Table 1, in which nearly all prior spinal cord doses remained below a conventional 50 Gy equivalent dose. Therefore, such a factor has to be deferred to future works. It is more likely for such factors to be explored for organs with milder complications than the spinal cord. In the meantime, we caution readers that the present model only applies when the prior dose was within tolerance limits, in accordance with the existing reirradiation guidelines,^3^, ^9^, ^16^ and a minimum time interval to reirradiation of at least 5 months.^3^, ^16^

### 4.2 Limitations and future studies

The biological basis for human recovery from radiation is currently not fully known, so the choice of recovery model is based on intuition and somewhat arbitrary. Future studies should explore potentially better models for all organs from head to toe—that is the goal of the proposed ReTEC project, but it would take many more datasets from many institutions to achieve.

It is known that myelopathy can occur decades after radiation therapy,^30^ but accurate incidences at such lengths of time are challenging to acquire. Therefore, conservatively, the search for *R_max_* was limited to 95%. Since the maximum likelihood algorithm reached that limit, it is plausible that the actual recovery might be greater than that at very long time intervals between courses. But we wanted to ensure that this initial model is conservative. Future studies are needed with more data at long time intervals between courses.

We began this project with a more thorough goal to include any dose‐fractionation in the model. However, for non‐SBRT data, the initially modeled recovery times were longer. Since we could not identify definite reasons for the differences, we decided to publish this first model using only patients who had at least one course of SBRT exposing the spinal cord, and to explore models of other fractionations in the near future. Potential reasons may include the larger volume of the spinal cord that is irradiated, older datasets with older technology, and reirradiation with lower biologically effective doses. However, the predominant reason could simply be the lack of published complete voxel‐wise datasets.

Philosophically, some may aim for lower risk in the reirradiation setting due to the additional complexity and uncertainty, whereas others may accept higher risk due to the gravity of the situation. This dilemma is beyond the scope of the present manuscript; our goal is merely to analyze reirradiation recovery within the context of dose response modeling and clinical examples, as in Figure 2, to at least convert reirradiation composite dose into an equivalent physical dose compatible with the de novo therapy dose, so that others may more accurately decide the benefit and risk tradeoffs.

The goal of the proposed ReTEC project is to overcome these limitations by systematically analyzing dose response models of published data to understand the effects of dose and recovery time on tissue toxicities, thereby refining dose‐time recovery factors in the reirradiation setting. Currently, there is insufficient published data to achieve this objective, but this new spinal cord recovery model provides the opportunity to propose the ReTEC project in a meaningful way. Several prospective reirradiation protocols are being conducted, such as Reirradiation Dose Escalation in Thoracic Cancers (REPAIR) NCT06558175 and the ReCare study NCT03818503;^5^ hopefully the data and results of these will be available to analyze as part of the ReTEC project. The Reirradiation Collaborative Group (ReCOG) project^31^ is currently defining practice guidelines, consensus statements, clinical tools, and workflows to improve reirradiation treatments and to facilitate the synthesis of multi‐site dose/volume/outcome data.^32^ However, at the start of the ReCOG project, it was decided that dose response modeling with recovery factors was beyond the scope because it would take too long, and indeed, by the time we are publishing the first human spinal cord recovery model to propose ReTEC, the ReCOG project is already well underway.^33^, ^34^, ^35^, ^36^

### 4.3 Reporting standards

For accurate NTCP modeling of reirradiation, dose‐time‐volume data of each course for each anatomic structure and the time interval between courses, as well as the baseline and follow‐up outcomes, for each patient individually included in the data set, are necessary. The number of fractions used for each patient is needed to properly estimate the best value and the uncertainty impact of α/β. Most papers do not report this information individually for each patient, which is the main limitation of all the QUANTEC / HyTEC / PENTEC papers. Preferably, the dose‐time‐volume information for each course would include the 3D Digital Imaging and Communications in Medicine (DICOM) dose distribution and contours so that composite plans and recovery factors could be performed per voxel, and to gain a complete understanding with accurate modeling, including bath‐and‐shower effects^26^ and other volume effects. When 3D DICOM is not available or feasible, it is important to note that the hot spots in each plan generally occur in different locations, so DVH cutpoint (Dx/Vx) dose descriptor values will be needed for each course, as well as for the composite, so that the summation of the courses can be verified.

Several concepts of time are important for reirradiation dose tolerance models: (1) there may be dose‐rate effects as a function of the delivery time of each fraction,^37^ (2) the fractionation regimen directly impacts the total number of elapsed days during a course of treatment, and is the basis of the typical definitions of BED, (3) the time interval between courses is needed to assess normal tissue recovery, (4) the timing of the onset and duration of patient symptoms is important because of late effects, necessitating that the dose‐response modeling be performed as a function of time,^38^ (5) the timing of local recurrences is important because we need to differentiate between toxicities that necessitate lower dose versus treatment failures which necessitate higher dose, and (6) the length of follow‐up and overall survival are needed to assess competing risks. The main focus of the present work is the time interval between courses, but we want to at least enumerate the other aspects of time because they will be important for improved recovery models in the future. Ideally, the ReTEC project would include all fractionations, but for the proposal, this first model included only patients reported to have SBRT utilized in at least one of the courses.

Accurate pooled models can only be achieved when each source dataset is published with clear and specific definitions. Particularly for each patient, it is essential to know the time interval between each course of treatment, the timing of complications, local control, survival, and the length of follow‐up. However, these definitions often vary and are not always clearly specified. For instance, the HyTEC pancreas tumor control paper^39^ needed the time interval between surgery and radiation, as well as the length of follow‐up. Some of the studies, however, measured time starting from study enrollment, or from the time of diagnosis, surgery, the beginning or end of chemotherapy, the time of first intervention, or the start or end of radiation, while others did not define time at all. For fair comparisons with other modalities, such as chemotherapy and surgery, it would be best to use the date of diagnosis as the starting time definition for overall survival. When authors want to use alternate definitions, like the last day of treatment, they should report using both definitions: an appendix could have tables of doses and outcomes, like survival times for each patient by several definitions. Like a Rosetta Stone, this would enable any necessary conversions for future studies. In the reirradiation models, we would like to use the recovery time interval from the end of each course to the beginning of the next course, but we acknowledge that definitions in the source papers vary and some are unclear, and we ask that future authors clearly define the time intervals used.

### 4.4 Differential dose tolerance threshold for different segments of neuraxis

It should be noted that different segments of the serial neuraxis may have different dose tolerances, analogous to differences among the hollow organs of the gastrointestinal tract. Even across the spinal cord proper, there may exist slightly differential radiosensitivity. Brainstem is known to have a higher dose threshold than the spinal cord, at least for small volumes.^40^ The brainstem actually has physical separation from the vertebral bodies of the spinal column; thus, the brainstem was easily excluded from our model. For the same reason, the sacral plexus, which has a higher dose threshold,^41^ was fairly accurately excluded from our analysis. Importantly, the spinal cord and cauda equina overlap at the upper lumbar spinal level around L1‐2 in a potentially different location for each patient. From this point, the cauda extends downward within the thecal sac that spans the entire spinal canal. Because the dosimetric information from the available data more frequently lacks segregation of spinal cord from the cauda, often thecal sac being used as the only defined spinal cord surrogate structure,^42^ our initial analysis will not separate out the cauda from the spinal cord. Especially this is a challenge in reirradiation studies because conventionally fractionated initial courses were often to a larger region with excess margin, potentially including a substantial portion of the adjacent vertebral body, which could span across L1‐2 more than an SBRT reirradiation plan typically would. Supplemental Table A2 summarizes the variations in spinal cord and the vertebral body levels treated in the source data used in this model, but without actual contours of the spinal cord at each level (cervical, thoracic, lumbar, sacral), it is not possible to stratify the model to this level of detail. This reminds us of the need for higher‐quality data and reporting standards, with full 3D voxel‐wise composite plans including contours of both spinal cord and cauda so they can be accurately separated to determine their dose tolerance differences.

### 4.5 Importance of adequate follow‐up

One critical requirement necessary to ensure the validity of the analysis and for accurate calculation of radiation myelopathy risk is adequate follow‐up. With SBRT, the median latent time to the development of radiation myelopathy in the absence of progression is reported to be 12 months after de novo treatment and 6 months after reirradiation.^3^ Radiation myelopathy in the control cohorts may not manifest over an insufficient duration of the follow‐up period. Conversely, some of the observed myelopathy events could actually be caused by the initial course instead of the reirradiation course. Sufficient follow‐up for analysis is limited by the fact that less than half of metastatic patients who undergo spine‐directed SBRT may be alive at one year.^43^ Death is a competing risk to the endpoint of radiation myelopathy in this patient population. With a lack of positive datapoints that would have otherwise been captured with a longer follow‐up, the radiation myelopathy risk could be underestimated. Future dose response studies, especially for reirradiation, where the time interval between courses is crucial, should incorporate these time kinetics actuarially as part of the modeling process.^38^

## 5 CONCLUSION

Determination of dose‐time recovery factors for the spinal cord to guide reirradiation dosing decision making is feasible based on this preliminary literature search and analysis. Our preliminary model suggests that existing guidelines^3^, ^9^, ^16^ might be safe for clinical practice of reirradiation, but many other unrecognized factors affect outcomes beyond what a model can predict, so it is entirely the responsibility of each clinician to make the decisions for their individual patients. Since this model was constructed only of max point doses from the paucity of data in the literature, we strongly advise not exceeding those existing guidelines and available dose constraints until more complete datasets have supported or refuted this model.

A large‐scale project like ReTEC will require the complete published datasets of many institutions to refine the results and NTCP models. Fifteen years ago, it was said that the sparsity of NTCP for SBRT data would make an endeavor like QUANTEC impossible for SBRT, but as it turned out, the existence of the HyTEC project itself might have helped to motivate the publication of about 90% of the current dose response models for the first course of radiosurgery. Likewise, sparsity of data should not be the reason to prevent this project; sparsity of solid data is the very reason that the proposed ReTEC project is necessary and timely, to help motivate publication of adequate reirradiation data sets, for improved understanding of radiation recovery.

## AUTHOR CONTRIBUTIONS

All authors have contributed to the work per ICMJE criteria: each author did have substantial contributions, drafted/reviewed the work, approved the final version, and is accountable for all aspects of the work. In addition, Dr. Grimm performed the dose response modeling using the DVH Evaluator software.

## CONFLICTS OF INTEREST DISCLOSURE

No funding was received for the present work; however, authors report the following relevant recent conflicts of interest:

Jimm Grimm designed and holds intellectual property rights to the DVH Evaluator software tool which is an FDA‐cleared product in commercial use, and which has been used for this analysis; Grants from Novocure, Accuray; Speaker honoraria / travel reimbursement from Accuray, PTW.

Scott Soltys reports Research Funding from Novocure; Consultant for Novocure; and Speaker Honoraria from Accuray and Brainlab.

Arjun Sahgal reports Honorarium for educational seminars from Elekta/Elekta AB, Varian, BrainLab, AstraZeneca, Seagen, Cerapedics, CarboFIX and Servier; Consultant for Elekta/Elekta AB and BrainLab; Research grants from Elekta/Elekta AB and Seagen; and Travel Expenses from Elekta/Elekta AB, BrainLab, and Cerapedics

Kristin J. Redmond reports Research funding: Accuray, Canon, Icotec, GammaTile, Travel support: Icotec, BrainLab, Patent pending: Canon, and Data safety monitoring board: BioMimetix.

John Austin Vargo serves on a committee for Elsevier Clinical Pathways.

Felix Ehret has received honoraria and travel support from ZAP Surgical Systems, Inc., and Accuray, Inc., and acknowledges research funding from the German Cancer Aid and Accuray, Inc., all unrelated to the submitted work.

Lawrence R. Kleinberg reports research support from BMS, Incyte, Novartis, and Novocure as well as honorarium for a study steering committee for Novocure.

John R. Adler is the CEO and an equity holder in Zap Surgical Systems, San Carlos CA.

No other authors have conflicts of interest relevant to the current work.

## Supporting information



Supporting information

## Data Availability

Data is available in the tables, figures, and supplemental files associated with the online journal article.

## Footnotes

1 D1cc = minimum dose to the “hottest” one cubic centimeter of spinal cord.
