# Supplementary material for: Reirradiation treatment effects in the clinic (ReTEC) proposal – proof of concept based on spinal cord dose tolerance for reirradiation with stereotactic body radiotherapy
Source: J Appl Clin Med Phys. 2026 Apr 9;27(4):e70557. doi: 10.1002/acm2.70557 (PMC13062923; doi:10.1002/acm2.70557)
Supplement: Supplementary file 1 — Supporting information [file ACM2-27-e70557-s001.docx]

**Appendix A**

Human spinal cord data: PubMed searches, literature review diagram, case study, additional tables.

PubMed searches performed in October 2024:

Fig. 1A: (radiosurgery OR hypofraction* OR SBRT OR SABR OR CyberKnife) AND (NTCP)

Fig. 1B: radiosurgery OR hypofraction* OR SBRT OR SABR OR CyberKnife

Fig. 1C: reirradiation OR re-irradiation

Table 1: (myelopathy OR neuropathy OR “spinal cord”) AND (reirradiation OR re-irradiation)

(Note: the neuropathy keyword was included initially to attempt a similar model for the optic nerve, but we did not find sufficient data for that yet.)

Fig. A1. Preferred Reporting Items for Systematic Reviews and Meta-Analyses (PRISMA) diagram of the literature search for dose response modeling. We found 13 of 362 articles that reported enough information to construct re-irradiation recovery dose response models for the spinal cord. Those articles also provided time-to-event, length of follow-up, and grade information, but since the model has only 6 myelopathy cases and four parameters to fit, it was deemed to be too sparse to also incorporate that information into the model. *Abbreviations*: Dmax = maximum point dose; NTCP = normal tissue complication probability; Dxcc = minimum dose to the “hottest” *x* cubic centimeters of spinal cord.

To benchmark against published limits, a case study was scaled to two different dose levels from the HyTEC Spinal Cord Table 4 (ref003), reproduced in Table A1 (Examples A&B). The descriptions of Examples A&B are in the Results section of the manuscript, and are shown in Figs. A2 and A3. This example case study is the only patient that had any dose scaling, and it was not used in the model.

Table A1. Reproduction of HyTEC Spinal Cord Table 4 (ref003), with permission. To explore the effects of the recovery model in a wide range of doses, the case study was rescaled to the highest prior dose row (Example A) and also to the second-to-the-lowest prior dose row (Example B).


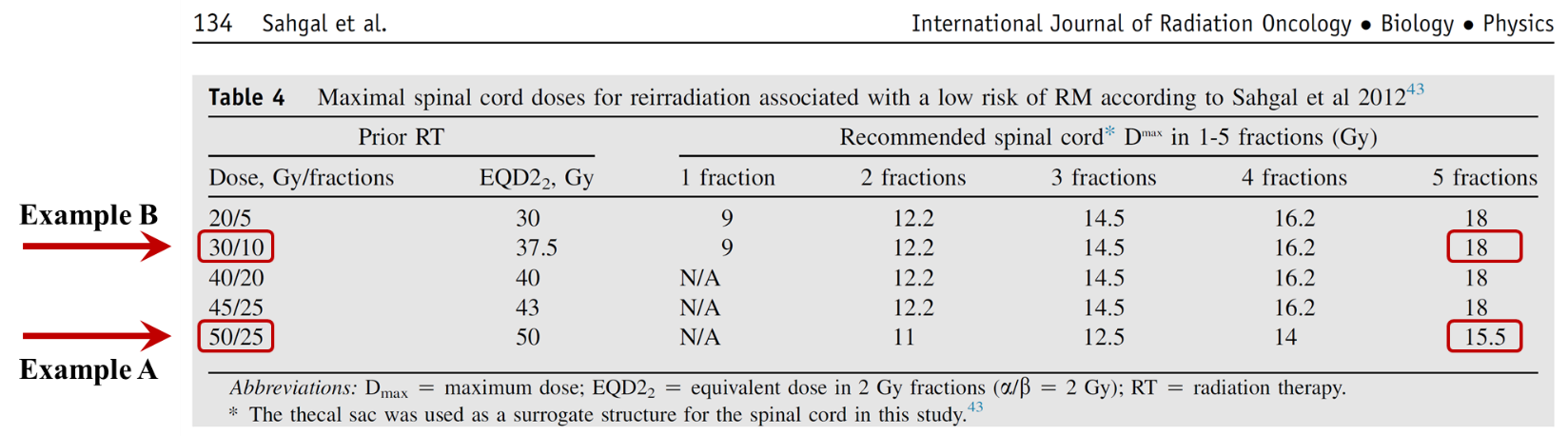


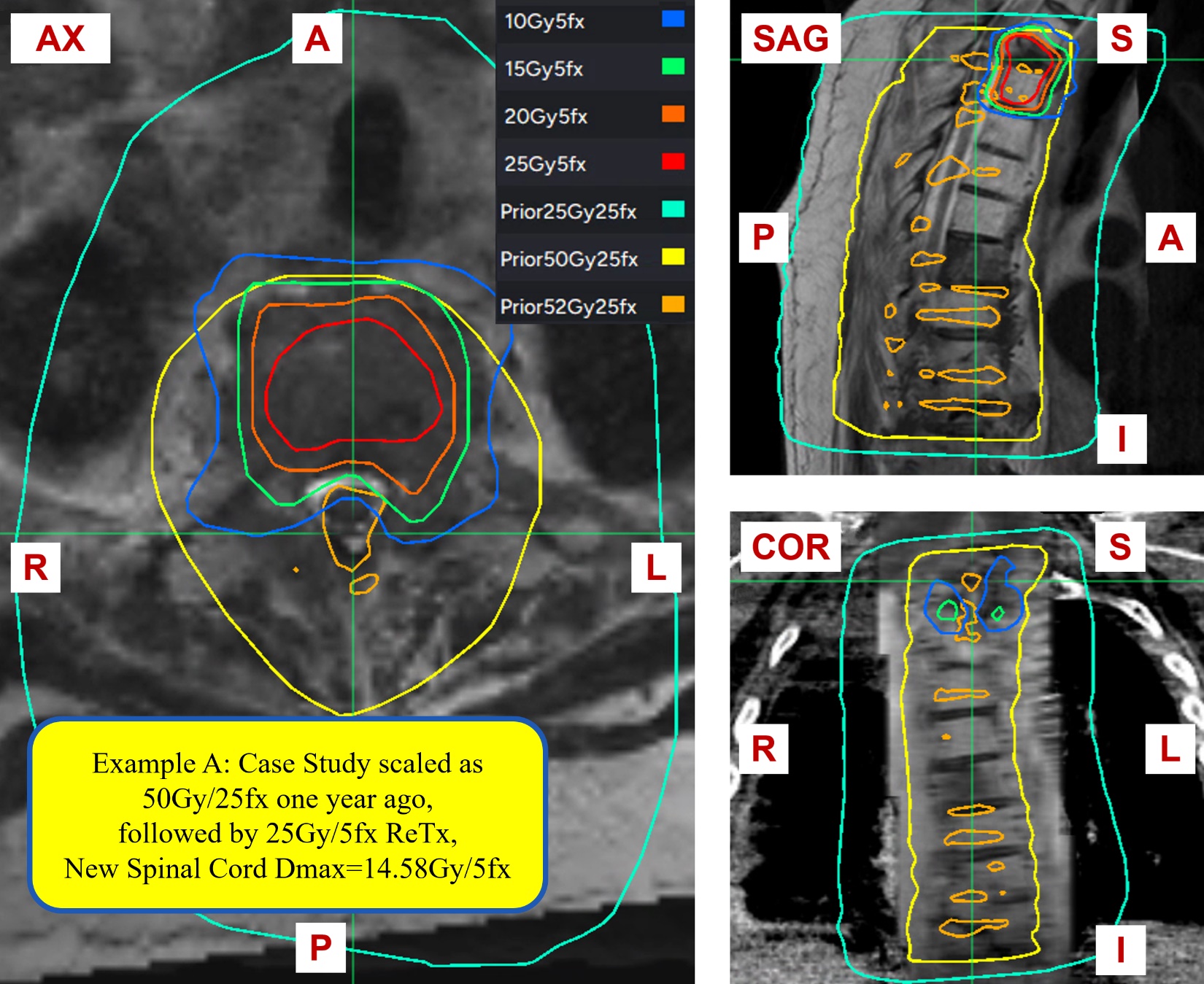


Fig. A2. Example A: The case study patient scaled to the highest prior dose row in Table 4 of the HyTEC spinal cord paper (ref003): 50 Gy in 25 fractions to T2-T12, a year before SBRT reirradiation of 25 Gy in 5 fractions to T2-T3, with spinal cord Dmax = 14.58 Gy (35.8 Gy BED_2_). The reirradiation cord dose was kept about 1Gy below the 15.5 Gy / 5 fraction limit because the conventional cord dose was 4.3% higher than the 50 Gy prescription (prior spinal cord Dmax=52.1 Gy, which corresponds to 106.3 Gy BED_2_). After the Nelson 2009 (ref009) recovery of 33% was applied, the total spinal cord point dose became 106.3 * (1-0.333) + 35.8 = 106.7 BED_2_, thus the corresponding 5-fraction equivalent dose was 28 Gy, which is 2 Gy below the TG-101 limit of 30 Gy in 5 fractions. From the figure, it can be seen that hotspots in the prior plan are near the high dose region of the spinal cord in the SBRT plan, so the 3D voxel-wise summation is within 0.2% of the point dose summation for this patient, but in general, the voxel-wise summations are more accurate.


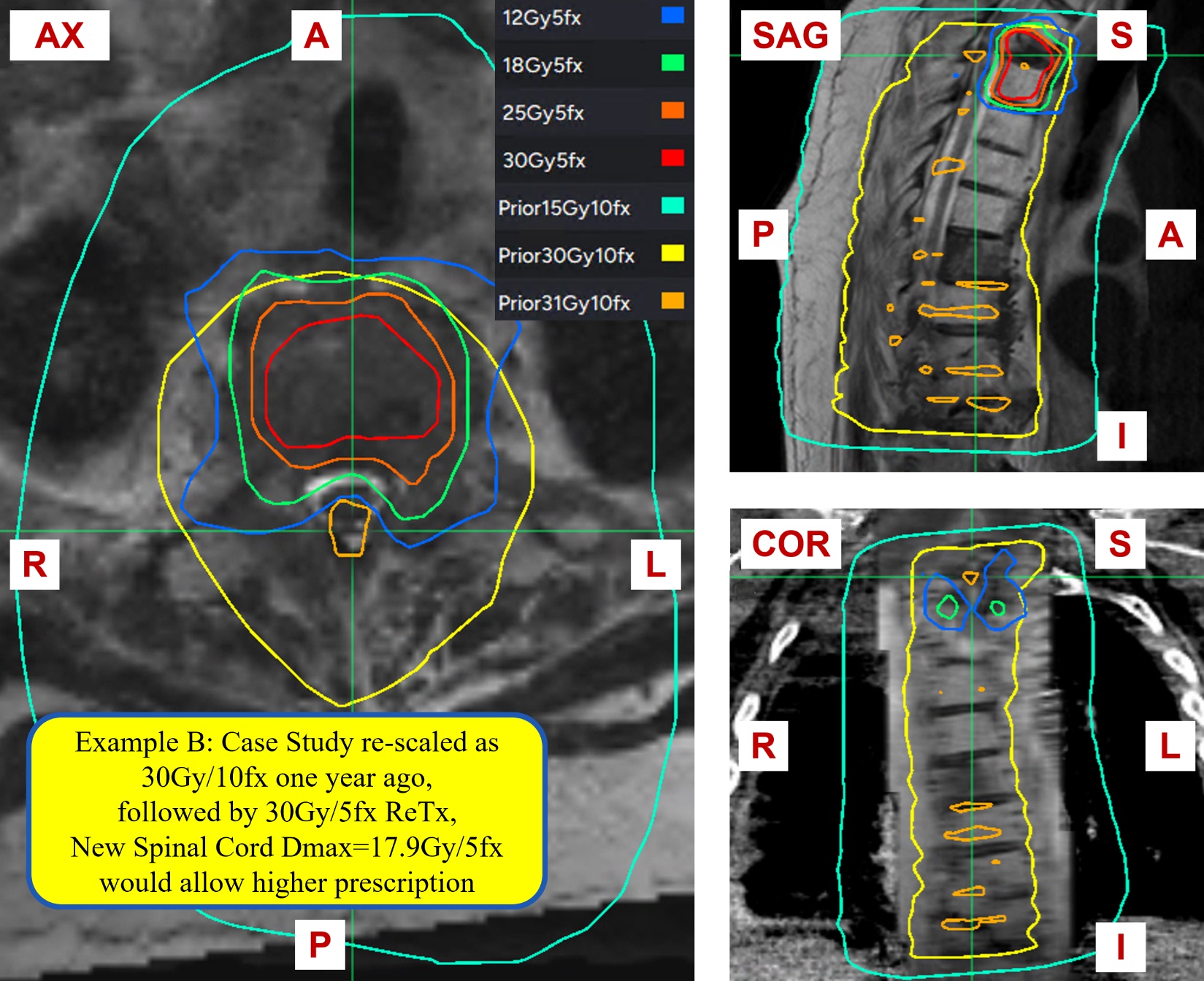


Fig. A3. Example B: The same case study patient, rescaled to the second-to-the-lowest prior dose row in Table 4 of the HyTEC spinal cord paper (ref003): 30 Gy in 10 fractions (spinal cord Dmax was 4.3% higher, 80.1 Gy BED_2_) one year ago, which enables a higher reirradiation SBRT prescription of 30 Gy in 5 fractions, with spinal cord Dmax = 17.9 Gy (49.9 Gy BED_2_). The total spinal cord Dmax, including recovery, was 80.1 * (1-0.333) + 49.9 = 103.3 Gy BED_2_ which corresponds to a 5-fraction equivalent dose of 27.5 Gy, a slightly lower total than Fig. A2 because of the lower prior dose. After refining the recovery models in the ReTEC project, it may be possible to fine-tune the limits, but just rescaling this case study a couple of different ways shows that the current reirradiation limits (ref003, ref016) provide very workable ranges.

Table A2. Comparison of definitions, treatment characteristics, and vertebral body treatment sites across the source data manuscripts. *Abbreviations*: NTCP = normal tissue complication probability; C, T, L, S = Cervical, Thoracic, Lumbar, Sacral Spine, respectively; CT = computed tomography; MRI = magnetic resonance imaging; T2 = long transverse relaxation time to visualize fluid; IG = image guidance; IMRT = intensity modulated radiation therapy; UCSF = University of California at San Francisco; PRV = planning at risk volume; CBCT = cone beam CT; CTCAE = Common Terminology Criteria for Adverse Events.

|  |  |  |  |  | **Number of Evaluable^a^ Cases** | | | |  |
| --- | --- | --- | --- | --- | --- | --- | --- | --- | --- |
| **First** | **Spinal Cord** | **Treatment Planning** |  | **Myelopathy Endpoint** | **Included in the NTCP model** | | | |  |
| **Author** | **Contour** | **Imaging and Contouring** | **Treatment** | **including** | **at Each Vertebral Body Level** | | | |  |
| **and Year** | **Definition** | **Technique** | **Types** | **Imaging Type** | **C** | **T** | **L** | **S** | **Notes** |
| Gwak 2005  (ref010) | Spinal cord | Myelopathy case had CT only. For the non-myelopathy cases, "MRI images were fused with CT images using Accufusion version 1.0 software (Accuray)" | CyberKnife | "Radiation-induced myelopathy was defined as the appearance of new upper motor neuron symptoms with MRI change of high signal intensity in the T2-weighted image with or without gadolinium enhancement in accordance with the irradiated level." | 3 | 0 | 0 |  |  |
| Gerszten 2006  (ref011) | Spinal cord; at and below cauda equina, spinal canal used | planning CT; CT and MRI used for diagnosis but MRI not specified for treatment planning. | CyberKnife | "no clinically detectable neurological signs that could be attributable to the acute or subacute radiation-induced cord damage. Posttreatment MRI revealed no changes suggestive of radiation-induced spinal cord injury." | 1 | 0 | 0 |  | "Exclusion criteria for radiosurgery treatment were: 1) evidence of overt spinal instability or 2) neurologic deficit resulting from bony compression of neural structures." |
| Gibbs 2007  (ref008) | Spinal cord with minimum margin | MRI-CT fusion. In rare cases a myelogram was used. | CyberKnife | "treatment-related severe myelopathy", not further defined | 0 | 2 | 0 |  |  |
| Parikh 2009  (ref012) | Spinal cord in patient with intramedullary cord lesion | MRI fused with planning CT | CyberKnife | "rare intermittent paresthesias in his fingers and toes" that improved after radiation as compared to baseline. "no other new neurological deficits" | 1 | 0 | 0 |  | Case report. Not spine but rather intramedullary cord metastasis |
| Choi 2010  (ref013) | Spinal cord | MRI fused with planning CT | CyberKnife | "Grade 4 spinal cord neurotoxicity" | 12 | 21 | 4 |  |  |
| Damast 2011  (ref014) | Spinal cord | Planning CT Myelogram or Fused MRI | Linac, Memorial Body Cradle, IG-IMRT | "no incidence of myelopathy", not further defined | 5 | 20 | 10 |  | Omitted 5 sacral cases |
| Nikolajek 2011  (ref015) | Spinal cord | MRI fused with planning CT | CyberKnife | "One patient with metastatic renal cell carcinoma developed a progressive complete paraparesis one year after the last treatment at lumbar level L3" | 7 | 24 | 23 |  | Omitted 7 sacral cases |
| Sahgal 2012  (ref016) | Thecal sac | "contoured thecal sac per UCSF practice (2–4), and each case was centrally reviewed" | 16 control cases treated with CyberKnife | "neurologic signs or symptoms consistent with myelopathy at the irradiated segment without evidence of progressive tumor" | 3 | 13 | 2 |  | The Gwak 2005 and Gibbs 2007 cases are excluded from this row, and reported in their own rows in this table. |
| Chang 2012  (ref017) | Spinal cord: "dura mater contoured at the level of the tumor"; Cauda equina: "the dural sac margin represented neural margin" | Not specified | CyberKnife | "no case of radiation myelopathy detected", not further defined | 28^b^ | | 9 |  | Omitted 14 sacral cases. |
| Wang 2014  (ref018) | Thecal sac, extended 6-6.25mm superior and inferior beyond tumor | MRI fused with planning CT | CyberKnife | "no radiation-induced myelopathy had occurred", not further defined | 4 | 5 | 3 |  |  |
| Thibault 2015  (ref019) | Cord + 1.5mm or thecal sac for cord PRV; thecal sac with no additional margin for cauda equina | Referenced earlier report that describes use of MRI fused with planning and CT myelogram if MRI was contraindicated | Elekta Synergy, CBCT, HexaPOD, BodyFIX | "no cases of myelopathy were observed", not further defined | 2 | 24 | 15 |  | Omitted 3 sacral cases |
| Zschaeck 2017  (ref020) | Spinal cord and canal both delineated separately if possible | MRI | Novalis, CyberKnife | CTCAE 3.0 myelitis grade 2 or higher | 0 | 1 | 1 |  |  |
| Ehret 2021  (ref021) | Spinal cord | MRI fused with planning CT | CyberKnife | "no myelopathies were observed", not further defined | 8 | 14 | 16 |  |  |
| Bentahila 2023  (ref022) | Spinal cord | MRI fused with planning CT | CyberKnife | CTCAE v5 | 0 | 1 | 0 |  | Case report |

^a^ The number of evaluable cases excludes sacrum, local failures, and patients without SBRT in any of the courses, as explained in the footnotes of Table 1

^b^ In this study it was not possible to differentiate C-Spine from T-Spine so they have been grouped together.

**Appendix B**

Comparison of the raised exponential recovery model to the Gompertzian, and matlab source code.

exp(-*k*1*(1-exp(-*k*2**t*)))

A) When *t* →∞ then:

exp(-*k*1) → 1-*R_max_*

so

*k*1=-ln(1-*R_max_*)

B) When *t* = *t_RH_* then:

0.5 = exp(-*k*1*(1-exp(-*k*2**t_RH_*)

ln(2)=*k*1*(1-exp(-*k*2**t_RH_*)

ln(1-ln(2)/*k*1)=-*k*2**t_RH_*

*k*2= -ln(1-ln(2)/*k*1)/*t_RH_*

Matlab source code for Equations (2)-(3):

t_RH=3.8962;

R_max=0.95;

t=1:240;

k=1./t_RH.*log((R_max)./(R_max-0.5));

RDE=1-R_max+R_max.*exp(-k.*t);

plot(t,RDE);


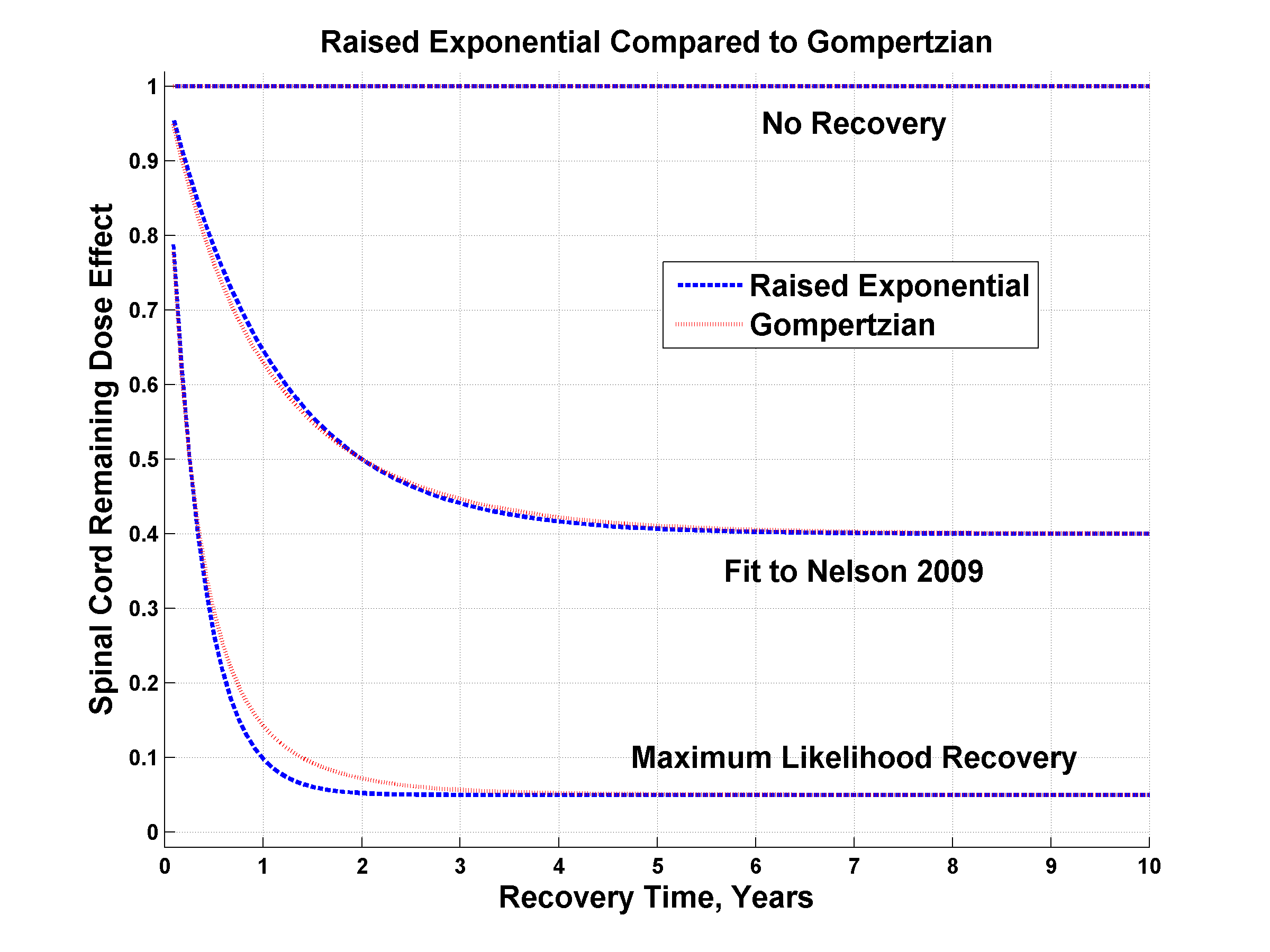


Fig. B1. Graphs of the raised exponential function as compared to the Gompertzian function for the three scenarios in Fig. 2.

**Appendix C**

Comparison of the raised exponential recovery model to Nelson 2009 values (ref009).


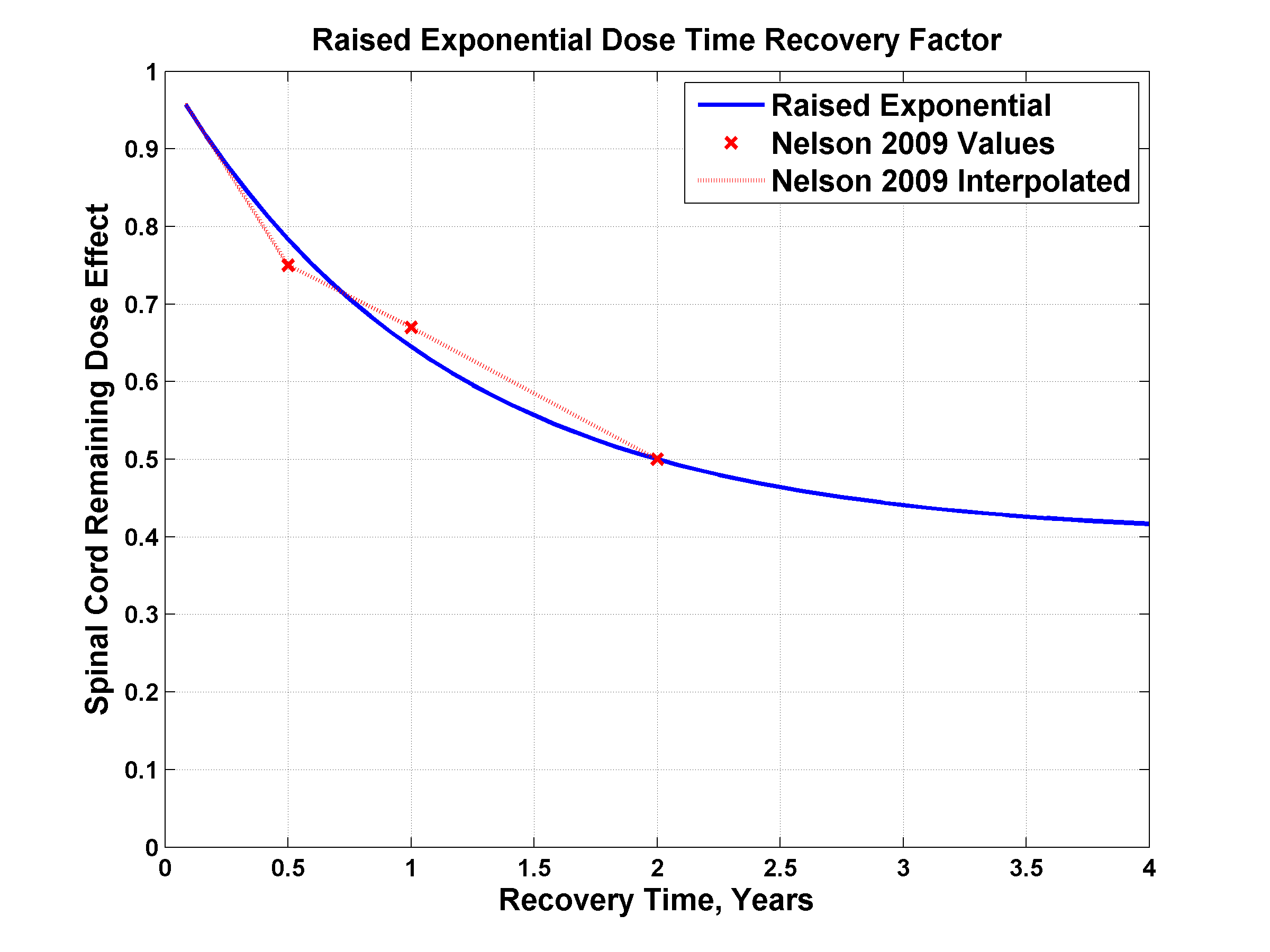


Fig. C1. Graphs of the raised exponential function fitted to the Nelson 2009 (ref009) dose recovery values of 25%, 33%, and 50% at 6 months, 1 year, and 2 years, respectively, as used for the scenario in Fig. 2(c-d).

**Appendix D**

Comparison of preclinical animal studies reoptimized with the newly designed raised exponential recovery model.

The new model in the body of this manuscript was created exclusively from the published human data in Appendix A and Table 1. Since it is the first known model of this type, it is important to compare it to something. Therefore, we used the same methods to reanalyze existing animal studies for verification and to gain insights.

The Wong 1997 study of 708 rats is analyzed with the new model in Fig. D1 (refD01, refD02). Whereas the Wong 1997 paper stratified the data into many specific sub-analyses, we instead applied the raised exponential recovery model to all of the 708 data points to form a single overall model. From that, a distinct advantage of animal models is clearly visible in Fig. D1, where it can be seen that the doses and times were systematically varied from less than 1% risk to beyond 99% risk, which is not possible in human data. This enables us to observe characteristics of the model in ideal circumstances, unlike in human data, which is usually skewed to very few responders for toxicity and very many responders for tumor control (refD03), consequently creating more challenging modeling circumstances.

According to the maximum likelihood fitted model in Fig. D1, the rat spinal cord *t_RH_* was 11 months, and *R_max_* was 85%. The Wong 1997 paper also observed about 50% recovery with an interval of 1 year, so the new model is reasonably consistent with the original result. No conversions were used to compare animal metabolism or tumor growth to humans, and large time intervals between courses are not available in animal data, so these values are not directly applicable clinically; nevertheless, it is interesting to see some similarities to Fig. 2.


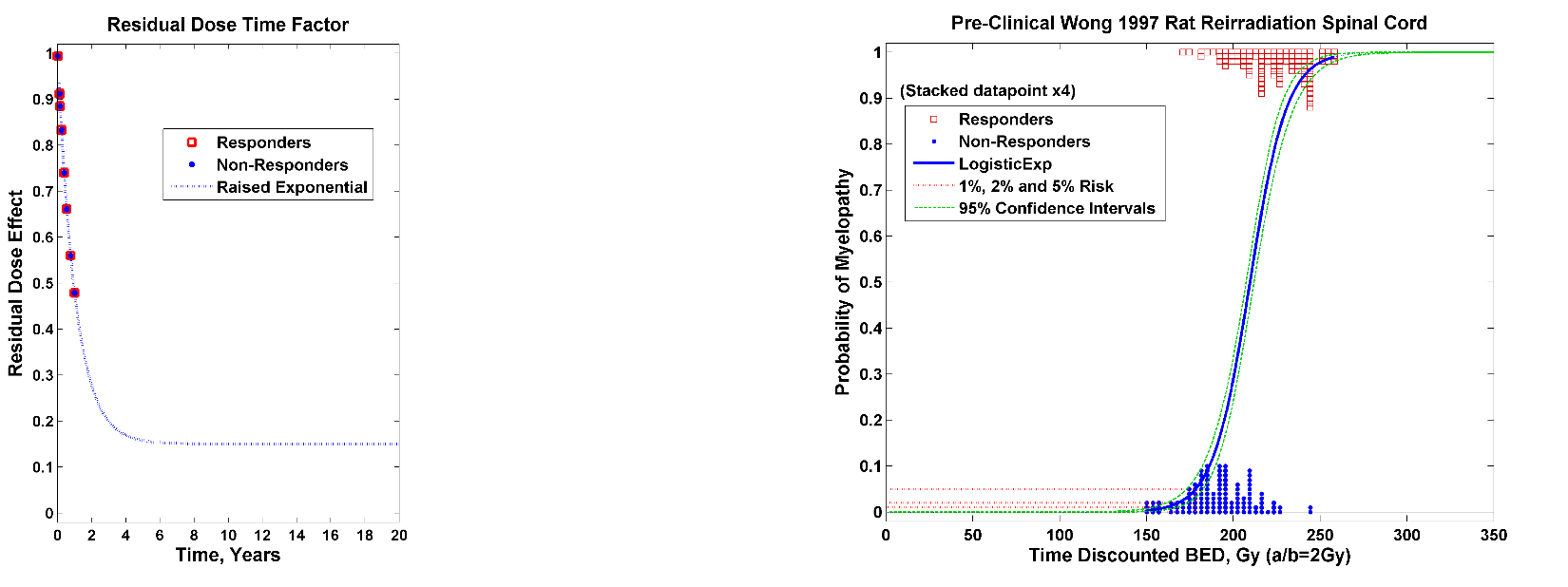


708 Female Fisher F344 Rats

Initial spinal cord dose

18 Gy/2 fx, 27 Gy/3 fx or 30.75 Gy/3 fx

Time interval 3 days to 1 year

Reirradiation spinal cord dose 4-18.5 Gy in one fraction

Fig. D1. Raised exponential dose-time recovery model applied to Wong 1997 pre-clinical animal study (refD01, refD02).

The Ang 2001 study of rhesus monkeys (refD04, refD02) was also reanalyzed using the raised exponential recovery model. All monkeys in Ang 1993 (refD05) and Ang 2001 (refD04) received 2.2 Gy per fraction in all courses, and the entirety of the analysis was done in terms of physical dose without BED conversions. The baseline for measuring recovery was a de novo model from single course irradiation of 70.4 Gy, 77.0 Gy, or 83.6 Gy in daily fractions of 2.2 Gy each, to 29 monkeys with evaluable follow-up, as reproduced in Fig. D2. Preliminary reirradiation studies of 15 evaluable monkeys were reported in Ang 1993, and the Ang 2001 paper supplemented those same dose regimens with 45 additional monkeys to complete a more accurate model; the sums of these that caused myelopathy are also overlaid onto Fig. D2.


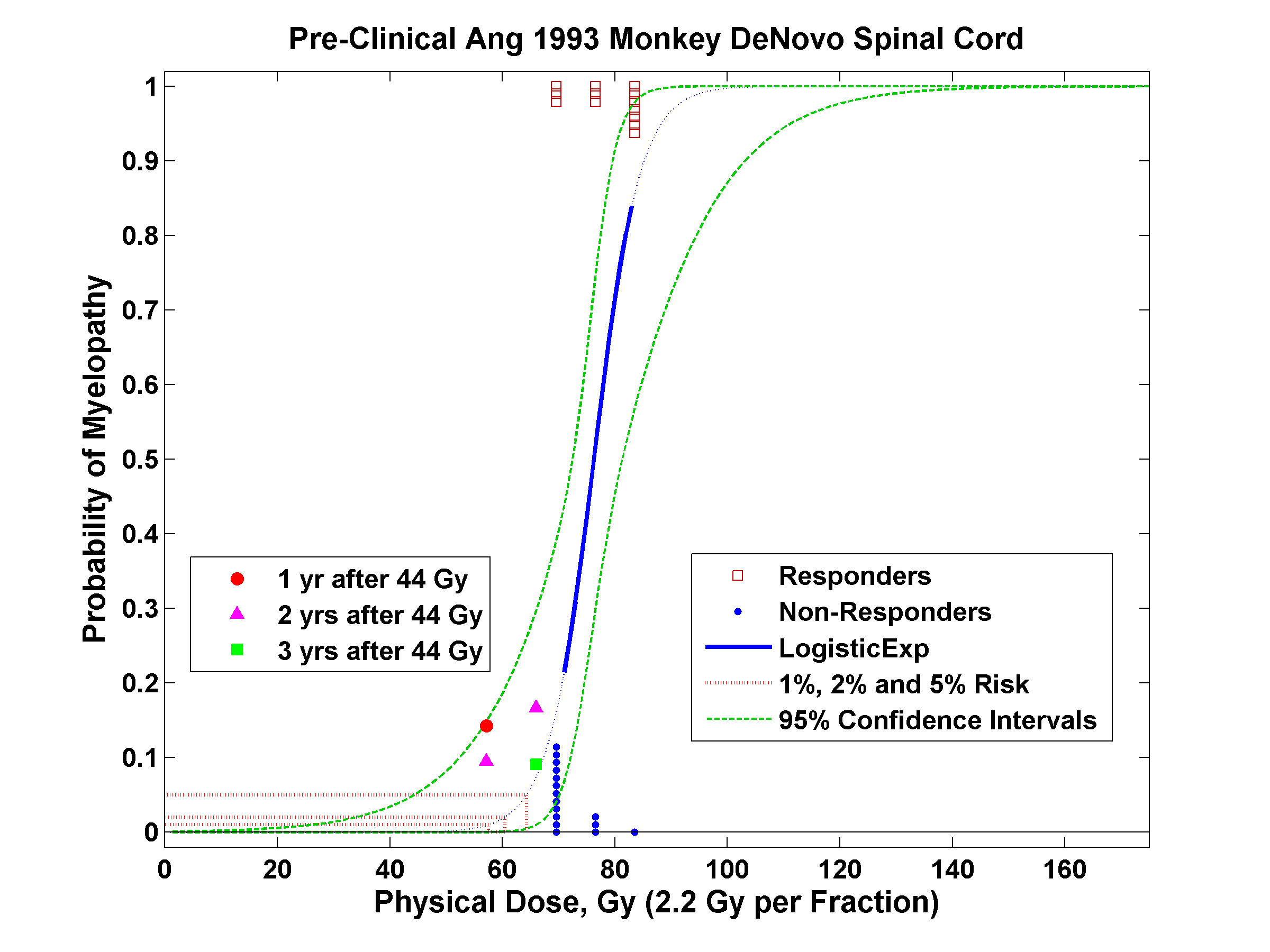


Fig. D2. Reproduced de novo monkey spinal cord dose tolerance model from Ang 1993 (refD05). Additionally, four reirradiation data points from Ang 2001 (refD04) are superimposed, with 44 Gy in 2.2 Gy per day followed by a reirradiation dose 1, 2, and 3 years afterward.

Table D1. Monkey Spinal Cord Recovery, in terms of physical dose in 2.2 Gy fractions without any biological conversions.

| **Initial** |  | **ReTx** | **Number** |  | **Crude** |  | **De novo** | **Inferred** | **Percentage** |
| --- | --- | --- | --- | --- | --- | --- | --- | --- | --- |
| **Spinal** | **Time** | **Spinal** | **of** | **Number** | **Risk** |  | **NTCP** | **Recovered** | **of 44 Gy** |
| **Cord** | **Interval,** | **Cord** | **Evaluable** | **of** | **of** | **Total** | **Corresp.** | **Dose,** | **that was** |
| **Dose, Gy** | **Months** | **Dose, Gy** | **Animals** | **AE** | **AE** | **Dose, Gy** | **Dose, Gy** | **Gy** | **Recovered** |
| 44 | 12 | 57.2 | 14 | 2 | 14.3% | 101.2 | 69.0 | 32.2 | 73.2% |
| 44 | 24 | 39.6 | 4 | 0 | - | 83.6 | - | - | - |
| 44 | 24 | 48.4 | 4 | 0 | - | 92.4 | - | - | - |
| 44 | 24 | 57.2 | 21 | 2 | 9.5% | 101.2 | 67.1 | 34.1 | 77.5% |
| 44 | 24 | 66 | 6 | 1 | 16.7% | 110 | 69.7 | 40.3 | 91.6% |
| 44 | 36 | 66 | 11 | 1 | 9.1% | 110 | 66.9 | 43.1 | 98.0% |

*Abbreviations:* ReTx = reirradiation; Gy = Gray; AE = adverse event (e.g., myelopathy); NTCP = normal tissue complication probability; Corresp. = corresponding.

All 60 evaluable monkeys from both Ang 1993 and Ang 2001 are consolidated into Table D1 in terms of physical dose. The four dose-time levels that resulted in myelopathy are plotted in Fig. D2. By comparing the crude risk of each of these four reirradiation data points to the de novo NTCP model in Fig. D2, we can infer the percentage of the initial 44 Gy that was recovered. For example, with a 12-month interval after 44 Gy, an additional 57.2 Gy, totaling 101.2 Gy, caused myelopathy in 2 of 14 monkeys, for a crude risk of 14.3%. However, in the de novo NTCP model, it would only take 69 Gy to reach a 14.3% risk. Since 101.2-69 = 32.2, we can infer that 32.2 Gy was “forgotten” or “recovered”, and that is 73.2% of the original 44 Gy. In this same manner, the inferred recovery of all four myelopathy data points is shown in Table D2.

Since the monkeys were irradiated in pre-set groups with the same conditions within each group, the recovery model can be fitted both as maximum likelihood and as least squares (Fig. D3). The least squares fitting was performed directly on the recovery model of equation (2) by calculating the mean squared error of the four myelopathy values in Table D1 as compared to all values of *R_max_* from 65% to 95% and all values of *t_RH_* from 1 week to 5 years with increments of 100 steps each. The minimum mean square error was parabolically interpolated from the result and then rounded, becoming *R_max_*=93% and *t_RH_*=6 months. The maximum likelihood fitting was performed by combining all 60 evaluable monkeys to form a single overall model, applying the recovery model of equation (2) for all the same range of *R_max_* from 65% to 95% and *t_RH_* from 1 week to 5 years with increments of 100 steps each. After parabolically interpolating the peak of the logistic model maximum likelihood over this range, the rounded result was *R_max_*=93% and *t_RH_*=6 months. Many expert modelers are currently testing this, so we defer all the goodness-of-fit tests and exact values to future work. For now, we just mention that the rounded values from both methods appear to be in general agreement. Some of the models in the Ang 2001 paper also showed 50% recovery with an interval of less than a year, so the new model is reasonably consistent with the original result.

In the first model of the Ang 2001 paper, "all of the curves were required to be parallel at the 50% incidence level," implying that the slope of the reirradiation NTCP model was constrained to be the same as the de novo NTCP model, and we used that same methodology in this Appendix. That constraint was not needed in the least squares method, so the fact that the maximum likelihood method with that constraint matches the more direct least squares method lends credibility to the approach. Furthermore, the 95% confidence intervals in Fig. D3 suggest that an unconstrained model would have a lower slope, which would be more hazardous if used clinically; the steeper slope serves as a safer warning to guide dose escalation. The 1% risk level of the human spinal cord model in Fig. 2(c-d) happened to match the de novo 1% risk level (ref003) fairly well without any such manipulations - it is possible that this indicates that neither the de novo nor the reirradiation human model has data at a sufficiently high dose to estimate the steepness of the true dose-response accurately. Thus, from the Ang 1993 and 2001 monkey datasets, we can verify the concept that missing high-dose data may cause the slope to be estimated less steep than reality, which should be a consideration when designing human dose escalation studies.


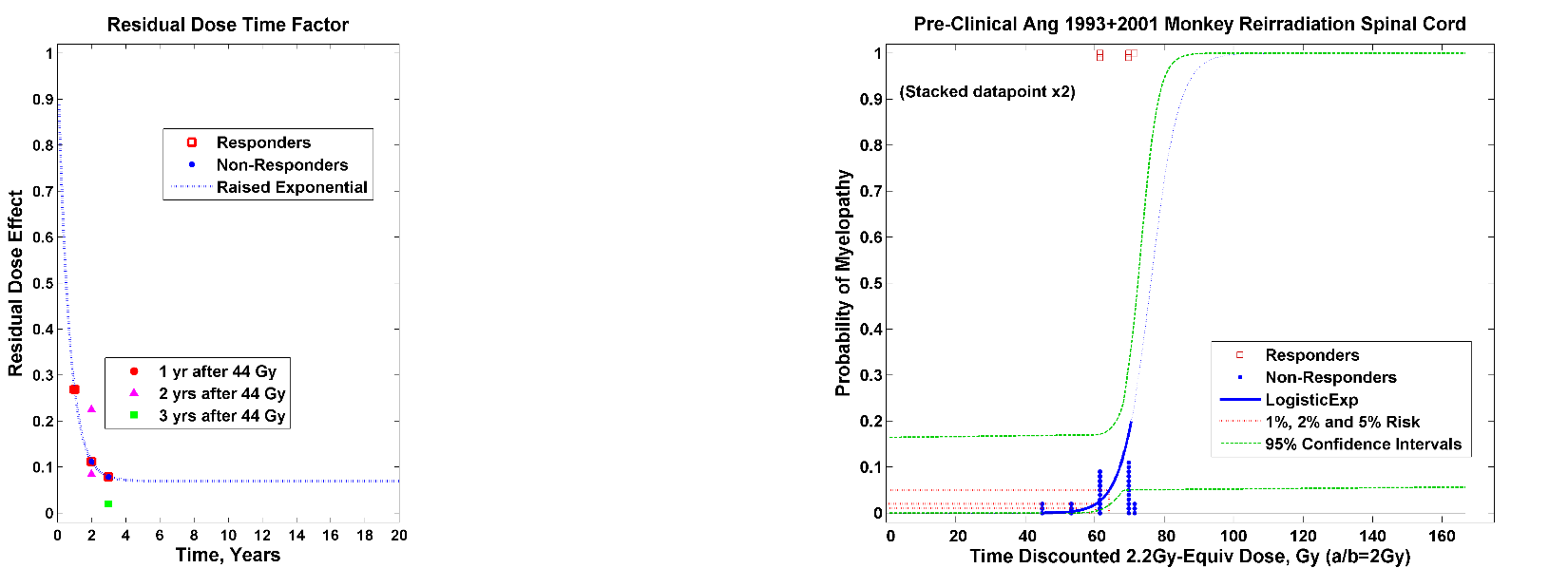


60 Evaluable Rhesus Monkeys

Initial spinal cord dose

44 Gy/20 fx

Time interval 1,2, or 3 years

Reirradiation spinal cord dose 39.6-66 Gy in 2.2 Gy per fraction

Fig. D3. Reproduced reirradiation monkey spinal cord dose tolerance model from Ang 2001 (refD04). Additionally, four reirradiation data points from Ang 1993 and Ang 2001 (refD05, refD04) are superimposed, with 44 Gy in 2.2 Gy per day followed by a reirradiation dose 1, 2, and 3 years afterward.

Since there are no other similar known human datasets currently available for comparison with the new model, we have used it to reanalyze existing animal models, and it achieved comparable results to the original publications (refD01, refD04, refD05). That should not be interpreted to mean that those animal models are valid for human clinical use, but instead, our goal in those comparisons was only to validate the functionality of the new reirradiation recovery modeling technique.

Appendix D1 References

refD01. Wong CS, Hao Y. Long-term recovery kinetics of radiation damage in rat spinal cord. Int J Radiat Oncol Biol Phys. 1997 Jan 1;37(1):171-9. doi: 10.1016/s0360-3016(96)00453-1. PMID: 9054893.

refD02. Schultheiss T. Radiation Myelopathy. Springer, Cham. pp1-240. doi: https://doi.org/10.1007/978-3-030-94658-6. ISBN: 978-3-030-94657-9. Jul 2022.

refD03. Moiseenko V, Marks LB, Grimm J, Jackson A, Milano MT, Hattangadi-Gluth JA, Huynh-Le MP, Pettersson N, Yorke E, El Naqa I. A Primer on Dose-Response Data Modeling in Radiation Therapy. Int J Radiat Oncol Biol Phys. 2021 May 1;110(1):11-20. doi: 10.1016/j.ijrobp.2020.11.020. Epub 2020 Dec 23. PMID: 33358230; PMCID: PMC9339232.

refD04. Ang KK, Jiang GL, Feng Y, Stephens LC, Tucker SL, Price RE. Extent and kinetics of recovery of occult spinal cord injury. Int J Radiat Oncol Biol Phys. 2001 Jul 15;50(4):1013-20. doi: 10.1016/s0360-3016(01)01599-1. PMID: 11429229.

refD05. Ang KK, Price RE, Stephens LC, Jiang GL, Feng Y, Schultheiss TE, Peters LJ. The tolerance of primate spinal cord to re-irradiation. Int J Radiat Oncol Biol Phys. 1993 Feb 15;25(3):459-64. doi: 10.1016/0360-3016(93)90067-6. PMID: 8436524.
